# L-carnitine ameliorated fatty liver in high-calorie diet/STZ-induced type 2 diabetic mice by improving mitochondrial function

**DOI:** 10.1186/1758-5996-3-31

**Published:** 2011-11-15

**Authors:** Yunqiu Xia, Qing Li, Weizhen Zhong, Jing Dong, Zhulin Wang, Chunbo Wang

**Affiliations:** 1 Center for Human Functional Experiment, Medical College, Qingdao University, Ningxia Road, Qingdao, China; 2 Class 5, Grade 2007, Medical College, Qingdao University, Ningxia Road, Qingdao, China; 3 Physiology Department of the Medical College, Qingdao University, Ningxia Road, Qingdao, China

**Keywords:** Type 2 Diabetes Mellitus, Carnitine, Nonalcoholic Fatty Liver Disease (NAFLD), Mitochondria

## Abstract

**Background:**

There are an increasing number of patients suffering from fatty liver caused by type 2 diabetes. We intended to study the preventive and therapeutic effect of L-carnitine (LC) on nonalcoholic fatty liver disease (NAFLD) in streptozotocin (STZ)-induced type 2 diabetic mice and to explore its possible mechanism.

**Methods:**

Thirty male Kungming mice were randomly divided into five groups: control group, diabetic group, pre-treatment group (125 mg/kg BW), low-dose (125 mg/kg BW) therapeutic group and high-dose (250 mg/kg BW) therapeutic group. The morphology of hepatocytes was observed by light and electron microscopy. LC and ALC (acetyl L-carnitine) concentrations in the liver were determined by high-performance liquid chromatography (HPLC). Moreover, liver weight, insulin levels and free fatty acid (FFA) and triglyceride (TG) levels in the liver and plasma were measured.

**Results:**

Average liver LC and ALC levels were 33.7% and 20% lower, respectively, in diabetic mice compared to control mice (P < 0.05). After preventive and therapeutic treatment with LC, less hepatocyte steatosis, clearer crista and fewer glycogen granules in the mitochondria were observed. Decreased liver weight, TG levels, and FFA concentrations (P < 0.05) in the liver were also observed after treatment with LC in diabetic mice. Moreover, liver LC and ALC levels increased upon treatment with LC, whereas the ratio of LC and ALC decreased significantly (P < 0.01).

**Conclusion:**

LC supplements ameliorated fatty liver in type 2 diabetic mice by increasing fatty acid oxidation and decreasing the LC/ALC ratio in the liver. Therefore, oral administration of LC protected mitochondrial function in liver.

## Introduction

Nonalcoholic fatty liver disease (NAFLD), one of the most common complications of type 2 diabetes, is characterized by an increase in fatty acids, triglycerides, and cholesterol levels [1] and fat accumulation in the liver. Among type 2 diabetes patients, 50%-70% of individuals were diagnosed with NAFLD; in obese patients, that value increases to 95% [2]. Recent studies have shown that NAFLD and insulin resistance [3] were involved in metabolic syndrome (MS), especially in fatty acid metabolic disorder, which primarily occurred in obese and type 2 diabetes patients [4]. Prolonged exposure to free fatty acids damages pancreatic β-cells and hepatocytes [5]. Furthermore, excessive fat accumulation in the liver damages mitochondria, which are the primary cellular sites for fatty acid utilization [6,7]. Current general therapies to treat the early stages of fatty liver disease include lifestyle modification approaches such as exercise and weight loss with diet. The goals of these strategies are to normalize aminotransferase levels and to reduce liver fat levels and inflammation. However, none of these strategies specifically improve mitochondrial function in type 2 diabetes patients. The aim of the current study was to find treatments that would improve mitochondrial function in fatty liver disease in type 2 diabetes patients.

L-Carnitine (L-β-hydroxy-γ-N-trimethylaminobutyric acid [8,9]) (LC) is present in the free or acyl-carnitine form in the plasma [10]. LC plays an important role in lipid metabolism; it acts an essential cofactor for the β-oxidation of fatty acids by facilitating the transport of long-chain fatty acids across the mitochondrial membrane as acyl-carnitine esters. It can activate carnitine palmityl transferase-1 (CPT-1), the key enzyme in fatty acid oxidation [11]. Furthermore, because LC shuttles acetyl groups from inside to outside the mitochondrial membrane, it increases the CoA-SH/acetyl-CoA ratio in the mitochondrion by forming Acetyl L-carnitine (ALC) with the help of carnitine acetyltransferase (CAT) [12,13], whose activity relieves inhibition of the PDH complex and increases glycometabolism [14-16]. According to a previous study, significantly reduced lactate plasma levels suggested that LC can also stimulate the activity of pyruvate dehydrogenase (PDH) [17], whose activity is deficient in type 2 diabetic patients [18]. These results demonstrated that LC can act as a carrier of acetyl groups from the mitochondria to the cytosol [15,19]. In addition, LC was defined as a biomarker to assess the function of the mitochondria [20]. Evidence showed that a combination of nutrients, including LC, can improve mitochondrial dysfunction in the liver of type 2 diabetic Goto-Kakizaki rats [21]. Recently, Karanth et al. proposed that LC supplements enhanced the activity of mitochondrial enzymes such as CPT-1 and the respiratory chain enzymes [22]. Based on the evidence described above, we explored the effects of LC on NAFLD caused by type 2 diabetes.

The earliest study of the effect of LC on steatohepatitis was performed by Bowyer et al. in 1988 [23]. They claimed that LC deficiency was not the major cause of liver steatosis. However, a recent study showed that LC played a role in reducing steatosis in patients with hepatitis C treated with IFN alpha and ribavirin [24]. LC supplements in patients with NASH (non-alcoholic steatohepatitis) greatly improved glucose plasma levels, lipid profiles and histological manifestations [25]. However, none of these studies focused on the effect of LC on NAFLD caused by type 2 diabetes, especially in diabetic mice.

The aim of the present study was to explore the preventive and therapeutic effect of LC on NAFLD in streptozotocin (STZ)- and diet-induced type 2 diabetic mice with regard to morphological and biochemical aspects. We also intended to determine whether its effect was due to an improvement in the mitochondrial function of hepatocytes to provide theoretical evidence for LC as a clinical therapy for NAFLD.

## Materials and methods

### Animals

Male Kunming SPF 3-week-old mice (Institute for Drug Control of Qingdao, China), weighing between 15 g and 17 g were used. They were fed a diet containing 59% basic mice feed, 20% sugar, 18% lard, and 3% egg yolk. They had free access to food and water in an animal room maintained at 22 ± 3°C. The protocols used for handling the mice were approved by the Qingdao University Center for human functional experiment of the medical college Animal Care Committee and were in accordance with the guidelines set by the National Institutes of Health *Guide for Care and Use of Laboratory Animals*.

The animals were randomly divided into five groups: a control group (only high-calorie diet), a diabetic group, a preventive treatment group (125 mg/kg, intragastric, i.g.) and two therapeutic groups (250 mg/kg, 125 mg/kg, i.g.). At the age of 6 and 9 weeks, two low doses of STZ (100 mg/kg, i.p., provided by Sigma) prepared in 0.1 N citrate buffer at pH 4.5 were given to mice in the diabetic, preventive and therapeutic groups. Age-matched control group mice were injected with NS (normal saline) alone. Mice with fasting blood glucose levels above 12 mmol/L and insulin levels within the average range of the control group were considered type 2 diabetic mice.

### Drugs

LC (provided by Northeast Pharmaceutical Factory, China) was dissolved in double distilled water. In the study of the preventive effect of LC on hepatopathy in type 2 diabetic mice, oral administration of LC (low-dose, 125 mg/kg, i.g.) began on the day the mice were provided the high-calorie diet (3 weeks of age). LC was administrated to the therapeutic groups after they had developed diabetes (9 weeks of age). The two therapeutic groups of diabetic mice were given different oral doses of LC, a high-dose (250 mg/kg, i.g.) and a low-dose (125 mg/kg, i.g.). All the administrations were given once a day until the mice were 12 weeks of age. In addition, LC was administrated to mice in the preventive and therapeutic groups 30 min before samples were collected. Mice in the control group were given the same volume of saline. (Table 1: experimental design)

**Table 1:** Experimental design

Group	3 weeks old	6 weeks old	9 weeks old
Control	HFD+NS	HFD+NS (CB)	HFD+NS (CB)
DM+NS	HFD+NS	HFD+NS (STZ)	HFD+NS (STZ)
Pre-treat	HFD+LC	HFD+LC (STZ)	HFD+LC (STZ)
DM+LC(L)	HFD+NS	HFD+NS (STZ)	HFD+LC (STZ)
DM+LC(H)	HFD+NS	HFD+NS (STZ)	HFD+LC (STZ)

Control: control group (n = 6), DM+NS: diabetic group (n = 6), pre-treatment: preventive group (n = 6), DM+LC (L): low-dose therapeutic group (125mg/kg, i.g.) (n = 6), DM+LC (H): high-dose therapeutic group (250mg/kg, i.g.) (n = 6). All administrations of LC were given orally. HFD: high-fat diet; STZ: streptozotocin; LC: L-carnitine; CB: citrate buffer. At the age of 6 and 9 weeks, mice were injected twice with STZ or CB, respectively.

### Preparation of plasma and liver homogenates

All mice were sacrificed at 12 weeks of age. Blood samples were collected via retro-orbital bleeding. Fresh livers were observed and weighed. Livers were homogenized, and 1 ml of supernatant was collected and stored at -20°C until analyzed.

### Liver and body weights

Body weights were measured once a week with an electric balance (Mettler Toledo, PL1501-S, Shanghai, China). Whole explanted livers were weighed on an electric analytical balance (Mettler Toledo, AL104, Shanghai, China). Relative liver weights (per body weight) were calculated (relative liver weight: liver weight/body weight×100%).

### Liver morphology

Samples were washed with phosphate-buffered saline and fixed with 4% paraformaldehyde. Longitudinal sections (10 μm) were cut in a microtome (model CM1900; Leica, Germany), placed on microscope slides and stained with oil red O. Image Pro Plus 6.0 was used to quantify the size of the lipid droplets. For electron microscopy analysis, liver apexes were processed according to routine procedures [26]. Ultrastructural changes in the liver were observed by transmission electron microscopy (TEM) (provided by JEOL, 1200EX).

### Measurement of triglyceride and FFA concentrations in the plasma and liver

Liver free fatty acid levels were estimated by a Cu-colorimetric method using a non-esterified fatty acid assay kit (provided by Applygen Technologies Inc. Beijing, China). The results are expressed in absorbance values. TG levels in the plasma and liver were determined by photoelectric colorimetry using an automatic biochemical analyzer (Saturno-300, provided by CRNOY S.R.I Company, Italy).

### LC and ALC concentrations in the liver

The concentrations of LC and ALC in the liver were analyzed by high-performance liquid chromatography (HPLC). This method was first established in our lab [27]. A Waters2695 High Performance Liquid Chromatography system (Waters, America) equipped with a Hypersil C18 column (5 μm particle size, 200 mm length, 4.6 mm internal diameter) and a Waters474 fluorescence detector (Waters, America) was used.

### Statistical analysis

The data are expressed as the mean ± SD. Statistically significant differences between groups were assessed by analysis of variance (one-way ANOVA), repeated measures and multivariate tests followed by LSD. A P value of less than 0.05 was considered statistically significant.

## Results

### Changes in blood glucose and plasma insulin levels

Blood glucose levels were 197% higher in the diabetic group than in the control group (control+NS 6.35 ± 0.63 mmol/L vs. DM+NS 18.9 ± 6.04 mmol/L, P = 0.001). Plasma insulin levels in diabetic mice were normal (control+NS 12.94 ± 0.91 mIU/L vs. DM+NS 11.56 ± 1.09 mIU/L, P = 0.503).

### Effects of LC on changes in body weight in STZ-treated mice

**Figure 1:**
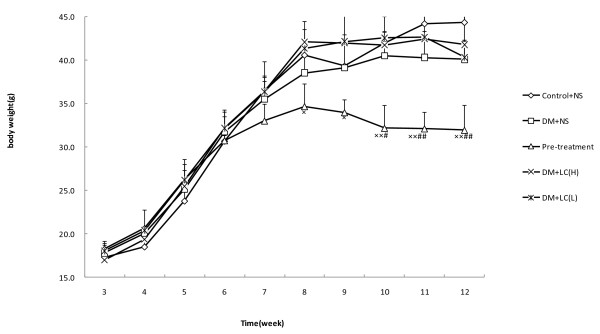
**Effects of LC on the changes in body weights of STZ-treated mice**. control: control group (n = 6), DM+NS: diabetic group (n = 4), pre-treatment: preventive group (n = 3), DM+LC (H): high-dose therapeutic group (n = 4), DM+LC (L): low-dose therapeutic group (n = 3). All values are expressed as the mean ± S.E. using the repeated measures method. *P < 0.05, **P < 0.01 (compared to control group), ^#^P < 0.05, ^##^P < 0.01 (compared to diabetic group).

At the 8th week, the average body weight of the preventive group was 14.6% lower than in the control group (control group 40.617 ± 2.919 g vs. preventive group 34.7 ± 2.571 g, P = 0.016). From the 10th week on, significant differences in body weight between the preventive and diabetic groups were apparent (P = 0.012). At the end of the 12th week, mice in the two therapeutic groups were heavier than those in the diabetic group (Figure 1).

### Lipid metabolism

All diabetic mice had higher liver FFA (+22.6%) and TG (+402.6%) levels, plasma TG levels (+225%), liver weight (+84.6%) and relative liver weight (+50%).

As shown in table 2, after 3 and 6 weeks of treatment with oral LC, liver FFA levels decreased in the pre-treatment, high-dose and low-dose therapeutic groups. There was no significant difference in FFA levels between these mice and mice in the control group (P>0.175). LC therapy also remarkably decreased liver TG levels (DM+NS vs. pre-treatment P < 0.01, DM+NS vs. high-dose P < 0.01, DM+NS vs. low-dose P < 0.05). Moreover, the mean plasma TG levels were 1.2 ± 0.1 mmol/L in the control group and 3.9 ± 2.3 mmol/L in the diabetic group. LC caused a decreased in plasma TG levels, especially in the high-dose and preventive treatment groups (high-dose therapeutic group 1.625 ± 0.399 mmol/L, preventive group 1.713 ± 0.636 mmol/L).

**Table 2:** Metabolic parameters

Variable	Control	DM+NS	Pre-treatment	DM+LC(L)	DM+LC(H)
Body Weight (g)	44.4 ± 3.4	40.1 ± 2.1	32.0 ± 2.8^××##^	40.3 ± 2.0	41.8 ± 5.6
Liver weight (g)	1.3 ± 0.3	2.4 ± 0.5**	2.0 ± 0.4*	1.9 ± 0.3*^#^	1.8 ± 0.2^#^
Relative liver weight	0.04 ± 0.01	0.06 ± 0.01**	0.06 ± 0.01	0.05 ± 0.00^#^	0.05 ± 0.00^##^
FFA in liver (L/g·cm)	1.7 ± 0.1	2.0 ± 0.3*	1.8 ± 0.2	1.9 ± 0.3	1.8 ± 0.1
TG in liver (mmol/L)	0.23 ± 0.04	1.16 ± 0.6**	0.34 ± 0.2^##^	0.55 ± 0.09^#^	0.48 ± 0.1^##^
TG in plasma (mmol/L)	1.2 ± 0.1	3.9 ± 2.3**	1.7 ± 0.6	2.2 ± 1.0	1.6 ± 0.4
LC in liver (μmol/L)	16.9 ± 3.4	11.2 ± 2.6*	12.1 ± 3.1	14.9 ± 1.9	12.6 ± 4.1
ALC in liver (μmol/L)	1.0 ± 0.2	0.8 ± 0.3	1.0 ± 0.7	1.4 ± 0.2	2.3 ± 0.9**^##^
LC/ALC	16.9 ± 4.7	16.6 ± 5.9	15.4 ± 6.6	11.3 ± 2.8	5.6 ± 1.5**^##^

Control: control group, DM+NS: diabetic group, pre-treatment: preventive group, DM+LC (L): low-dose therapeutic group, DM+LC (H): high-dose therapeutic group. All values are expressed as the mean ± S.E. using the repeated measures method. *P < 0.05, **P < 0.01 (compared to control group), ^#^P < 0.05, ^##^P < 0.01 (compared to diabetic group).

The average liver weight in the diabetic group was 44.6% higher than in the control group (table 2) (control 0.0439 ± 0.0050 vs. DM+NS 0.0635 ± 0.0103, P < 0.01). After administrating LC for prevention and treatment purposes, the liver weight significantly decreased. After treatment with LC, the relative liver weight also decreased, both in the high-dose therapeutic group (0.0489 ± 0.0020, P < 0.01 vs. diabetic group) and in the low-dose therapeutic group (0.0544 ± 0.0031, P < 0.05 vs. diabetic group). Furthermore, the effect of LC on liver weight was dose dependent.

### LC and ALC system

Mean LC and ALC levels were 33.7% and 20% lower in the diabetic group than in the control group, respectively (LC P = 0.033) (table 2). Mice in the preventive and therapeutic groups expressed higher levels of LC than those in the diabetic group, which were not significantly different than those of the control group. In total, the exogenous administration of LC could compensate for the deficiency of both LC and ALC in type 2 diabetic mice. Interestingly, average LC concentrations in the high-dose therapeutic group were lower than in the low-dose therapeutic group, whereas the high-dose therapeutic group expressed the highest ALC levels among the five groups (table 2). There were no significant differences in the LC/ALC ratio between the control and diabetic groups (Control+NS 16.9455 ± 4.7270 μmol/L, DM+NS 16.5659 ± 5.9218 μmol/L), whereas the ratio decreased after LC treatment. Among all the therapeutic groups, the ratio in the high-dose group was the lowest. The differences in the LC/ALC ratio between high-dose group and control and diabetic groups were both significant (P < 0.01).

### Morphological changes in the liver

The size of the livers in the diabetic mice was generally larger than in control mice. In general, the livers of diabetic mice were yellow in color with numerous particles on the surface and were much harder than normal livers. After treatment with LC, the size of the livers decreased, and the surfaces became smoother.

**Figure 2:**
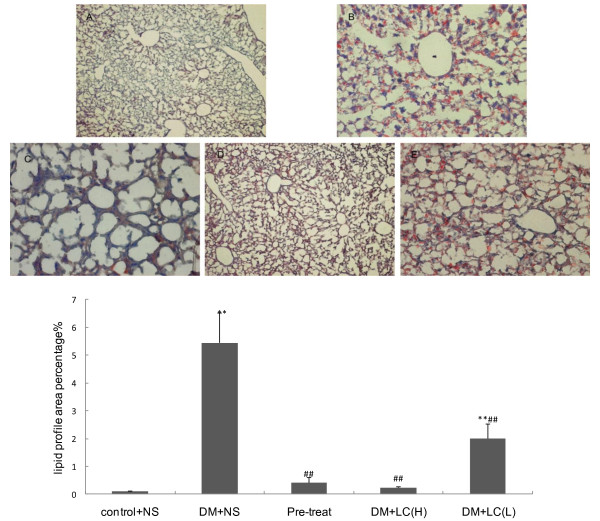
**LC's effect on liver sections stained by oil red O (×100 times)**. ***a, Oil red O images ***A. control group, B. diabetic group, C. preventive group, D. high-dose therapeutic group, E. low-dose therapeutic group. Red points in the image indicate lipid droplets stained by oil red O. ***b, Lipid profile area percentage ***Control+NS: control group (n = 3), DM+NS: diabetic group (n = 5), pre-treat: preventive group (n = 5), DM+LC (H): high-dose therapeutic group (n = 3), DM+LC (L): low-dose therapeutic group (n = 6). All values are expressed as the mean ± S.E. using the repeated measures method. **P < 0.01 (compared to control group), ^##^P < 0.01 (compared to diabetic group).

To analyze fat deposits in liver cells, liver sections were stained with oil red O. Obvious liver fatty degeneration and piecemeal necrosis of hepatocytes were observed in the diabetic group by LM (Figure 2-a). There were many red lipid deposits (stained by red oil O) in the hepatocytes of the diabetic group. As shown in Figure 2-I, the number of lipid deposits decreased with increasing doses of LC, especially in the preventive and high-dose therapeutic groups. Images from these two groups are very similar to those of the control group. As shown in Figure 2-II, the percentage of the lipid profile area to total area in diabetic mice was 5.44%, which is consistent with a clinical diagnosis of NAFLD [28]. Although the lipid area in the low-dose therapeutic mice was still fairly high (2.0 ± 0.52%), it was significantly lower than in the diabetic mice (P < 0.01). With the increasing dose of LC or the extension of treatment time, the lipid area percentage (Figure 2-b) decreased by 95.8% in the high-dose therapeutic group and 92.6% in pre-treatment group. There was no significant difference in lipid area percentage between mice in the LC treatment groups and the control group.

**Figure 3:**
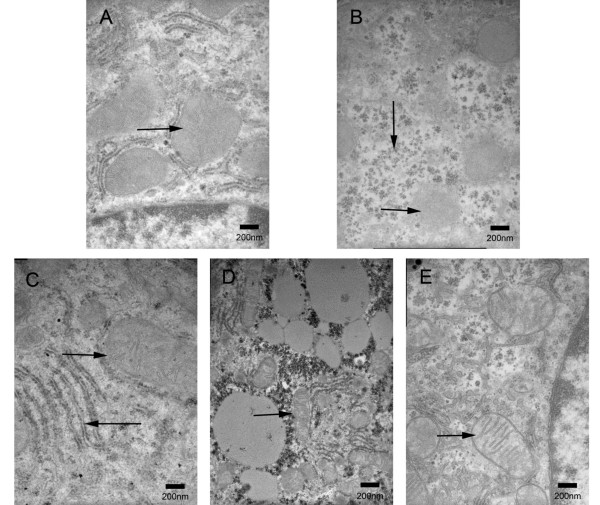
**Effect of LC on the liver by electron microscopy ***(×30K)*****. A. control group, B. diabetic group, C. preventive group, D. high-dose therapeutic group, E. low-dose therapeutic group. Mitochondrial (→), glycogen granules (↓), rough endoplasmic reticulum (←)

Obvious mitochondrial swelling, crista disorder and an increase in electron density were observed by TEM in the diabetic group (Figure 3B) compared to the control group (Figure 3A). In addition, there were more glycogen granules in the cytoplasm in the diabetic group, which is a characteristic of type 2 diabetes. Morphological damage of the mitochondria recovered after administration of LC. The LC groups possessed clearer crista (Figure 3C-E) and rough endoplasmic reticulum (Figure 3C) with dense granules.

## Discussion

### Model

Results from another study in our lab clearly indicated that we successfully created type 2 diabetic mice through a combination of a high-calorie diet and two low-dose STZ injections. Although the insulin levels of the diabetic mice were normal, the blood glucose levels were high. These results were consistent with characteristics of type 2 diabetes [29]. To verify the diagnosis of NAFLD, a liver biopsy, which is the gold standard [28], was performed. The biopsy revealed that the lipid area was above 5% *(*Figure 2-b*)*. Moreover, liver TG and FFA levels of diabetic mice were increased. All of these results are consistent with characteristics of type 2 diabetes: high glucose, insulin resistance, hyperlipidemia and obesity. Therefore, type 2 diabetes can be induced in mice by a combination of a high-calorie diet and two low-dose STZ injections to study the pathophysiological changes caused by diabetes or to evaluate the therapeutic effects of LC on fatty liver in type 2 diabetes.

### Morphological changes

The prevalence of NAFLD is high in obesity, diabetes, high-calorie diet and hyperlipidemia [30]. Our research confirmed the characteristics of NAFLD in diabetic mice. The gross appearance of the liver improved upon treatment with LC; the liver decreased in size and appeared brighter and less greasy. These changes were partly observed by quantifying the lipid droplets by LM (Figure 2-b). Therefore, it could be concluded that LC improved steatosis of the liver in type 2 diabetic mice. There was no obvious fibrosis in these images, suggesting that the diabetic mice in our study only suffered from fatty liver and had not yet developed cirrhosis. Therefore, this process was reversible. In addition, there were large areas of glycogen granules in the cytoplasm of diabetic mice, which is the electron microscopy feature of type 2 diabetes. Large numbers of granules attached to the rough endoplasmic reticulum in the preventive group, also suggested that cell function significantly improved upon administration of LC. To ensure the therapeutic effect of LC from a general perspective, body weight and relative liver weight values were evaluated. These results suggest that LC stimulated fat metabolism and improved mitochondrial function in the liver.

### Mechanisms of liver dysfunction caused by type 2 diabetes mellitus

**Figure 4:**
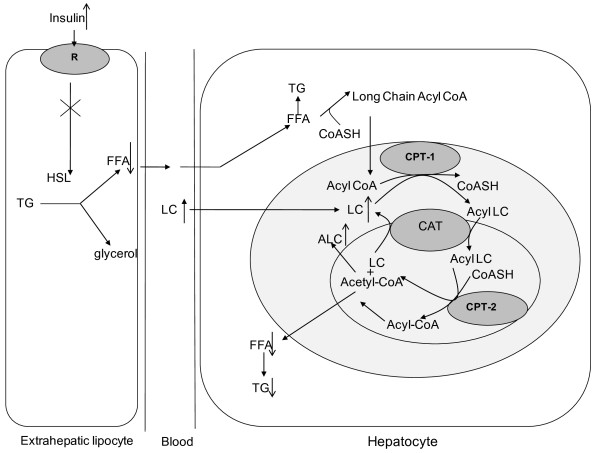
**Effect of LC on lipid metabolism in the liver and peripheral adipocytes in STZ-treated mice**. Acyl-CoA: long chain acyl CoA, CPT-1: carnitine palmitoyltransferase-1, CAT: carnitine/acylcarnitine translocase, HSL: hormone-sensitive lipase.

It was hypothesized that in type 2 diabetes mellitus, there was a shift in substrate utilization from carbohydrates to lipids [31]. From what has been discussed above, we knew that type 2 diabetic mice had disorders in both glucose and lipid metabolism. Glucose metabolism in diabetic mice was insufficient, which could relieve the suppression of lipolysis. The lack of suppression of lipolysis increased the influx of fatty acids to the liver, leading to increased free fatty acid and triglyceride levels in the liver (table 2). The peripheral fat storage was then consumed, and the liver weight and relative liver weight increased. After large amounts of FFAs enter the liver, β-oxidation of fatty acids was stimulated, further inhibiting glycolysis [32]. Moreover, evidence showed that high levels of FFA could stimulate gluconeogenesis. At the same time, increased FFA and TG levels could increase the burden of the liver [33]. Although the activity of CPT-1, which is the rate-limiting enzyme in fatty acid oxidation, was elevated when large amounts of FFA enter the liver [11], the deficiency of LC in type 2 diabetes limits its activity. Therefore, the activity of CPT-1 is not sufficient to handle the elevated transport of fatty acids into the liver. All of these factors lead to excess accumulation of hepatic fat, including FFAs and TGs [34]. It was reported that TG stored in non-adipocyte cells led to cellular damage as a result of their lipotoxicity [35]. This process is shown in Figure 4.

### The effects of LC

LC plays a critical role in fatty acid oxidation of energy regulation [36,37]. It serves as a carrier to facilitate the transport of long-chain fatty acids through the mitochondrial membrane and to undertake free fatty acid β-oxidation. Experimental evidence suggested that this step is a rate-limiting step in fatty acid oxidation. Diabetic mice had decreased liver LC levels (table 2), which is consistent with a previous study in which diabetic rats had significantly lower serum and liver LC levels than normal rats [38]. Administrating LC through i.g. compensated for the deficiency of LC and ALC in the liver. As a result, fatty acid β-oxidation increased [39], and lipid accumulation in the liver decreased. Elevation of fatty acid β-oxidation decreased fat synthesis. Therefore, the amount of TG transported from the liver into the plasma decreased. Plasma insulin concentrations were significantly elevated after different doses of LC, which is consistent with other studies in our lab. A previous study showed that insulin inhibited the activity of HSL (hormone-sensitive triglyceride lipase), the key enzyme in lipolysis (Figure 4). Therefore, the amount of FFAs transported from the peripheral fat to the liver decreased. In this way, the decrease in body weight in diabetic mice could be prevented. It is interesting to note that if LC was administrated before the mice developed diabetes, it prevented weight gain in diabetic mice from an early stage. This indicates that the obesity burden could be prevented. Therefore, preventive administration of LC to high-calorie diet-treated mice maintained the energy balance and resisted the effect of STZ. Overall, LC helped lighten the burden of the liver and improve the symptoms of NAFLD caused by type 2 diabetes. LC could also relieve the harm of high TG levels to the cardiovascular system and could therefore be beneficial to diabetic patients. Moreover, when body weight and liver lipid concentrations were controlled at relatively low levels, blood glucose levels were low in mice in the pre-treatment group (≤12 mmol/L). This helped mice fed a high-calorie diet resist the effects of STZ. Therefore, LC should be administrated in the early stages of diabetes.

Our study revealed that the ratio of LC to ALC decreased in the liver after LC administration even if both LC and ALC levels were markedly increased, which has not been observed before. According to a previous study, LC increases the CoA/acetyl-CoA ratio in the mitochondria by converting to ALC through the activity of CAT [12,13]. This process demonstrated that LC acted as a carrier of acetyl groups from the mitochondria to the cytosol [15,19]. In our study, ALC levels increased more in the high-dose treatment group, but LC concentrations in the low-dose therapeutic group were much higher than in the high-dose group. This result confirmed our hypothesis that exogenous LC supplements could shift the balance of LC + acetyl-CoA ⇌ ALC+ CoA to the right. Therefore, the concentrations of ALC and CoA were elevated. Previous data have shown that CoA was required for β-oxidation and other energy metabolism pathways [16]. Therefore, LC helped to decrease the accumulation of acetyl-CoA in the mitochondrial matrix. For this reason, both glucose and lipid metabolism could be stimulated. In another study, excessive acetyl-CoA was the substrate of lipogenesis [40]. Therefore, LC could also inhibit TG produce in liver by decreasing acetyl-CoA levels. Furthermore, plasma TG transported from liver was decreased.

### Liver function improvement

Disorders such as liver disease, defects in fatty acid metabolism, and the administration of pharmacological agents (e.g., pivampicillin or valproic acid) can cause secondary LC deficiency [41,42]. In our study, elevated FFA and TG accumulation causes lipotoxicity in hepatocytes, which could disrupt liver function. Mitochondrial dysfunction due to oxidative damage was previously detected in type 2 diabetes [21]. This dysfunction referred to a loss of capacity to synthesize LC and other proteins such as apolipoprotein and other useful factors. This aggravated damage to the liver and other organs. Exogenous administration of LC could compensate for this deficiency, which led to a beneficial cycle. Recently, DA Rossignol and colleagues used LC as a mitochondrial biomarker [20]. LC was indicated to be an essential factor in maintaining mitochondrial function. LC helped restore protein synthesis in mitochondrial membranes and showed an anti-oxidative effect [21,43]. The protective effect of LC on mitochondria was confirmed morphologically by EM (Figure 3). In conclusion, LC had an important mitochondrial detoxification effect on hepatocytes and other cells [41]. It improved symptoms of NAFLD and might improve metabolic conditions in type 2 diabetes mellitus.

## Conclusions

Type 2 diabetes induced by a high-calorie diet and two low-dose STZ injections in mice resulted in decreased liver LC contents. LC supplements improved mitochondrial function in the liver by accelerating the transport of free fatty acids into the mitochondria and regulating the matrix ratio of LC to ALC. LC might be an effective liver protective medicine for delaying the progression of type 2 diabetes mellitus complications.

## List of abbreviations

NAFLD: nonalcoholic fatty liver disease; LC: L-carnitine; ALC: acetyl L-carnitine; STZ: streptozotocin; NASH: non-alcoholic steatohepatitis; LM: light microscopy; EM: electron microscopy; FFA: free fatty acid; TG: triglyceride; HPLC: high-performance liquid chromatography; CPT-1: carnitine palmityl transferase-1; CAT: carnitine acetyltransferase; PDH: pyruvate dehydrogenase; DM: diabetes mellitus; NS: normal saline.

## Competing interests

The authors declare that they have no competing interests.

## Authors' contributions

YQX performed the high-performance liquid chromatography experiments and helped draft the manuscript. QL participated in the design of the experiments, carried out the study and drafted the manuscript. JD participated in the design of the experiments, revised the manuscript critically and gave final approval of the version to be published. ZLW participated in the design of the experiments and analyzed and interpreted the data. WZZ participated in the acquisition and analysis of data. CBW participated in the design and coordination of experiments. All authors read and approved the final manuscript.
